# Adherence is associated with a favorable outcome after lung transplantation

**DOI:** 10.1371/journal.pone.0226167

**Published:** 2019-12-17

**Authors:** Anna Bertram, Jan Fuge, Hendrik Suhling, Igor Tudorache, Axel Haverich, Tobias Welte, Jens Gottlieb

**Affiliations:** 1 Department of Nephrology and Hypertension, Hannover Medical School, Hannover, Germany; 2 Department of Respiratory Medicine, Hannover Medical School, Hannover, Germany; 3 Biomedical Research in Endstage and Obstructive Lung Disease Hannover (BREATH), Member of the German Center for Lung Research (DZL), Gießen, Germany; 4 Department of Cardiothoracic, Transplantation and Vascular Surgery, Hannover Medical School, Hannover, Germany; University of Michigan, UNITED STATES

## Abstract

Non-adherence to therapy is associated with impaired outcome in solid organ allograft recipients. Outcome data are limited after lung transplantation. In a single-center cohort study, adherence was assessed in 427 patients undergoing lung transplantation from 2010 to 2013. Objective criteria of adherence were judged by health care workers on every visit on a five item Likert scale including trough level monitoring, home spirometry and contact with an overall rating of adherence between 0 and 100%. Cut-off values for good vs. suboptimal adherence were defined retrospectively. Primary outcome was allograft survival, secondary outcomes were patient survival, prevalence of chronic lung allograft dysfunction, hospitalizations, renal function and quality of life. Follow-up ended on 31^st^ December 2018. Median adherence was 86% on 6,623 visits, this cut-off was used as a discriminator between good and suboptimal adherers. Patients with good adherence within the first three years showed better 5-year allograft (74% vs. 60%, p = 0.003) and patient survival (79% vs. 64%, p<0.001) and lower prevalence of chronic allograft dysfunction (33% vs. 45%, p = 0.011) after 5 years compared to patients with suboptimal adherence. A multidimensional adherence score proved to be a simple tool to assess adherence in clinical practice. Suboptimal adherence was associated with impaired outcome in lung transplant patients.

## Introduction

Lung transplantation (LTx) is an important therapeutic option in end stage pulmonary diseases, such as pulmonary fibrosis, emphysema, cystic fibrosis (CF), or pulmonary hypertension. Long-term allograft survival is limited by the development of chronic lung allograft dysfunction (CLAD), malignancy, infections, and comorbidities[1,2].

Non-adherence to therapy has been associated with impaired outcome in solid organ transplantation[3–5]. The assessment of adherence is a major challenge with potential dishonesty of patients’ being only one issue[6,7]. Adherence can be estimated by health care workers, with use of patients’ self-reports[8] and other instruments. Most publications focus on adherence to immunosuppressants, assessed with electronic medication event monitoring systems (MEMS), self-reports, or surrogate parameters like therapeutic drug monitoring[9,10]. Recently, non-adherence with immunosuppressive medication was associated with impaired survival of lung transplant patients in a large US registry analysis[11]. We have previously published the association of non-adherence with home spirometry and chronic lung allograft dysfunction (CLAD) in LTx recipients[12]. Other factors, such as health awareness, lifestyle or regular contact to the transplant center, might also influence outcome and may be useful in evaluating patient adherence. In order to assess adherence in LTx patients, we used a scoring system of five different indicators of adherence, completed by health care workers at every visit in the outpatient clinic. We hypothesized that good adherence assessed with this score is associated with allograft survival. Here we introduce our adherence score and analyze its potential predictive power on patient outcome.

## Methods

### Study design

We performed a single center retrospective cohort study. Hannover Medical School is an active LTx center and is following more than 1,000 patients in a specialized outpatient clinic. An adherence scoring system rated by transplant coordinators was developed and introduced in 2009 and since then used in all LTx outpatients on every visit.

All adult patients receiving first LTx between January 1^st^ 2010 and December 31^st^ 2013 that entered follow-up care in our outpatient clinic were included in this analysis. No other selection criteria were applied, so a selection bias should be excluded.

The study was performed in accordance with the ethical guidelines of the 1975 declaration of Helsinki. All patients provided informed consent prior to transplantation allowing the use of their data for scientific purposes, approved by the Ethics Committee of Hannover Medical School. According to the principles of our Ethics Committee, additional approval was not necessary, as data acquisition was retrospective and observational, data were anonymized and the study relied on measurements as part of routine care.

Primary outcome was allograft survival. Secondary outcomes were patient survival, prevalence of CLAD, hospitalizations within the first year after transplantation, renal function after 5 years, and quality of life within the first three years after transplantation. Spirometry was performed according to American Thoracic Society/European Respiratory Society guidelines. CLAD was defined as forced expiratory volume in 1 second (FEV1) <80% in relation to the baseline FEV1, defined as the average of the two highest measurements obtained at least 3 weeks apart during the postoperative course. Restrictive allograft syndrome (RAS) was defined as an additional decline in total lung capacity to <80% of baseline or significant opacities on thoracic CT scan after exclusion of other causes[13,14]. Patients were rated as having pure Bronchiolitis obliterans syndrome (BOS) if CLAD criteria were fulfilled but not criteria of RAS.

### Routine follow-up

Standard maintenance immunosuppression consisted of a triple drug regimen including calcineurin inhibitor (CNI), prednisolone and mycophenolate mofetil. Patients were instructed to use home spirometry on a daily basis and to send blood samples for immunosuppressant levels on a defined schedule (intervals 1–4 weeks) to the center’s central laboratory. Patients were encouraged to seek contact immediately in case of impairment in home spirometry, infections or other problems.

At each appointment in the outpatient clinic history, physical exam, spirometry and laboratory tests including immunosuppressant levels were performed. Surveillance bronchoscopy with bronchoalveolar lavage and transbronchial biopsy was performed at month 1, 3, 6 and 12 and whenever clinically indicated. Patients rated quality of life on a visual analogue scale (EQ-5D[15]) on every visit.

The follow-up period ended on December 31^th^ 2018.

### Adherence score

A three level Likert scale with five items was used (Table 1). For each item, three levels (not/partially/completely fulfilled; translated to 0/10/20% subscale) were assigned, so the sum of the five items resulted in a total adherence score of 0–100%. Items were chosen by being objectively measurable and distinct categories of adherence based on normal distribution and interim results of LTx outpatients in a training period of 3 months in early 2009 and levels were based on previously published cut-offs[12].

**Table 1:** Adherence evaluation tool.

Category	Good adherence (20%)	Moderate adherence (10%)	Suboptimal adherence (0%)
**Health perception**	Complete medication knowledge, full prophylaxis	Not fulfilling good or suboptimal criteria	Tobacco/drug abuse, inconsistent medication knowledge, poor diabetic control (HbA1c > 9%), sunbeds
**Home spirometry, frequency**	> 80% of recommended measurements	Between 50 and 80% of recommended measurements	< 50% of recommended measurements
**Contact**	Patient initiated contacts, call backs on messages	Not fulfilling good or suboptimal criteria	Missed appointments, inability to contact, emergency symptoms on routine visits
**Nutrition, Exercise**	Regular exercise, BMI between 18.5 and 25	Not fulfilling good or suboptimal criteria	No exercise, BMI < 17 or > 30
**Trough levels**	More than two third in target range	Between one and two third in target range	Less than one third in target range

Tool to evaluate the patients’ adherence using 5 different items on an outpatient clinic visit. Scores are summed up (0–100%).

Scores were assigned by transplant coordinators and discussed with physicians during daily team meetings, depending on the respective values and/or patient behavior at each visit. The scores were entered prospectively in a clinical and research database. The assignment of all subscores was necessary in order to obtain complete scores without missing values.

Smoking was ascertained by patients’ disclosure or by elevated cotinine values in blood samples. Cotinine levels were checked on clinical suspicion or when HbCO was ≥ 2% in arterial blood gas analysis. Poor diabetic control in diabetics was defined by highly elevated hemoglobin A1c levels (> 9%). All patients were instructed in the correct daily use of the home spirometry device in order to detect changes in FEV1 and allograft dysfunction early[16]. FEV1 is displayed on the monitor, and values are compared to individual best values. The spirometer is equipped with an alert system in case of declining FEV1. At each visit at the outpatient clinic, FEV1 values are transferred from the spirometer memory board to a database. Adherence in the category home spirometry was rated according to the frequency of use as previously published[12]. Contact was rated depending on how consequent patients kept their appointments or sought contact with the transplant center in case of medical problems such as worsening of home spirometry. An unexcused missed appointment resulted in a rating as suboptimal adherence at the next visit. Regularity of physical exercise was assessed by self-reports (regular physical exercise yes/no; which kind of exercise depending on individual capacity). Adherence to exercise is therefore not dependent on physical fitness. Body mass index (BMI) calculated from body weight and height (kg/m^2^) was displayed in the clinical database. All patients were individually instructed how to normalize BMI and all underwent a post-transplant in-patient rehabilitation with instruction to exercise regularly. CNI target ranges of 40 ng/ml for cyclosporine and 4 ng/ml for tacrolimus were defined for all patients (e.g., cyclosporine 50–90 ng/ml more than 2 years after LTx). Adherence was rated depending on the percentage of trough levels of the last 10 measurements within target range displayed in the database.

Adherence problems and results were discussed with the patients during visits. In case of suboptimal immunosuppressant levels, medication dosage was adjusted and potential influencers in co-medication and lifestyle were identified. The indication, correct handling and intake of immunosuppressive drugs and co-medication was repeatedly explained by health care workers including electronic educational programs[17]. The importance of lifestyle factors was regularly explained prior to and after transplantation by transplant coordinators and physicians. Negative impact of substance abuse, excessive exposition with UV radiation or poorly regulated diabetes were discussed with patients. Smoking, alcohol or drug cessation programs were offered. The purpose of daily home spirometry was explained repeatedly. Contact problems were discussed during visits. Patients were encouraged to regularly perform physical exercises or to take part in medical programs at fitness centers depending on their capabilities. For patients in poor condition, rehabilitation programs were recommended.

### Statistical analysis

The IBM SPSS Statistics (version 23.0, IBM Corp., Armonk, NY) and STATA (version 13.0, StataCorp, College Station, TX) programs were used for data analysis. Categorical variables are presented as numbers (n) or percentages (%), continuous variables as means ± standard deviation (SD) or medians and interquartile ranges (IQR), unless indicated otherwise.

Receiver operating characteristic (ROC) curves were drawn and additionally the median as well as tertiles and quartiles were used to find a cut-off value to predict allograft survival. Categorical variables were compared by chi-square test or Fisher exact test. Continuous variables were compared by Mann-Whitney U-test or two-sided paired t-test as appropriate. Kaplan-Meier estimates on allograft, patient and CLAD-free survival were made for patient groups using the median. The group estimates were compared by log-rank test. Stratified analysis by diagnosis was conducted using the above-mentioned methods for comparing subgroups. All reported p-values are two-sided unless indicated otherwise; p-values <0.05 were considered statistically significant.

## Results

Between January 2010 and December 2013, 519 patients (including 35 children) received LTx in our program. Of 460 adult patients with primary LTx, 427 reached the outpatient clinic, and adherence scores were assessed for them on a regular basis. Thirty-three patients did not reach outpatient care, because they died during initial hospitalization, and they were not included in the study. Patient demographics are shown in Table 2. The median follow-up period was 5.9 years (IQR 4.1–7.3). In total, 6,623 scores were analyzed in 427 patients, and 43,280 CNI trough levels obtained during visits and by sent-in blood samples, corresponding to 33.8 serum levels per patient-year, were included in our analysis. Based on an assumed daily use of home spirometry, more than 400,000 measurements were collected and analyzed. In total, 179 patients experienced graft loss: 151 died during follow-up and 28 received re-transplantation, of which 6 died.

**Table 2:** Patient demographics.

Characteristics	All Patients (n = 427)	Patients with good adherence (n = 213)	Patients with suboptimal adherence (n = 214)
**Age**—Median (IQR)	56.2 (43.3–62.3)	56.5 (46.4–62.6)	56.1 (40.3–62.3)
**Sex**—n (%)			
- Male	228 (53)	124 (58)	104 (49)
- Female	199 (47)	89 (42)	110 (51)
**LTx procedure**—n (%)			
- Bilateral	405 (95)	209 (98)	196 (92)
- Unilateral	8 (2)	2 (1)	6 (3)
- heart lung	13 (3)	1 (1)	12 (6)
- other combined	1 (0)	1 (1)	0 (0)
**Age at last Transplantation**—Median (IQR)	52 (39–58)	52 (43–58)	52 (36–58)
**Follow up**—Median (IQR)	3.8 (2.8–4.6)	3.8 (2.7–4.6)	3.7 (2.9–4.8)
**Diagnosis**—n (%)			
- cystic fibrosis	91 (21)	46 (22)	45 (21)
- pulmonary fibrosis	125 (29)	65 (31)	60 (28)
- emphysema	135 (32)	65 (32)	66 (31)
- Other	76(18)	33 (16)	43 (20)
			
**BOS Stage at last follow-up**—n (%)			
- BOS 0/0p	301 (71)	151 (71)	123 (58)
- BOS 1	25 (6)	18 (9)	27 (13)
- BOS 2	21 (5)	11 (5)	14 (7)
- BOS 3	80 (19)	33 (16)	50 (23)
**Visits within 1st Year**—Median (IQR)	8 (6–10)	8 (6–11)	7 (6–10)
**Visits within 2nd Year**—Median (IQR)	4 (3–6)	4 (3–6)	4 (3–6)
**Visits within 3rd Year**—Median (IQR)	3 (2–4)	3 (2–4)	3 (2–4)
**Adherence**—Median (IQR)	86 (79–90)	90 (88–92)	79 (74–83)

All numeric variables are shown as median with 25 and 75% interquartile range (IQR); all categorical variables are shown as n (%). BOS–bronchiolitis obliterans syndrome. LTx–lung transplantation.

### Adherence scores

**Fig 1:**
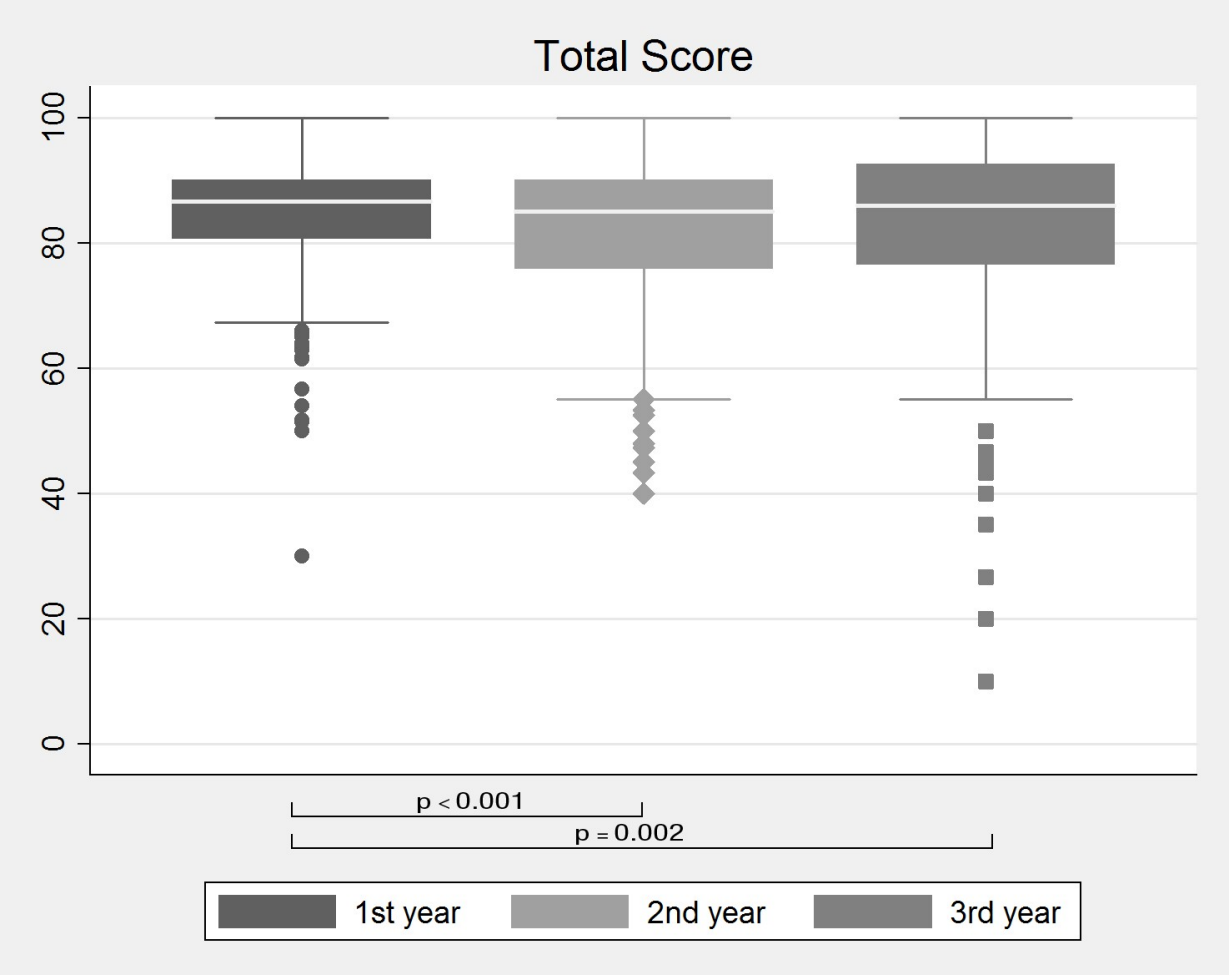
Adherence score comparison over time. Median combined adherence scores with IQR and outliers within the first three years after LTx.

Adherence in the first three postoperative years was rated good in general. Median adherence within the first three years after LTx was 86% (IQR 79–90%) with slightly better values in the first (median 87%; IQR 81–90%) as compared to the second (85%; IQR 76–90%) or third year (86%; IQR 77–93%) (Fig 1). There was no difference between male and female patients and no correlation between adherence and patient age (Table 2).

**Fig 2:**
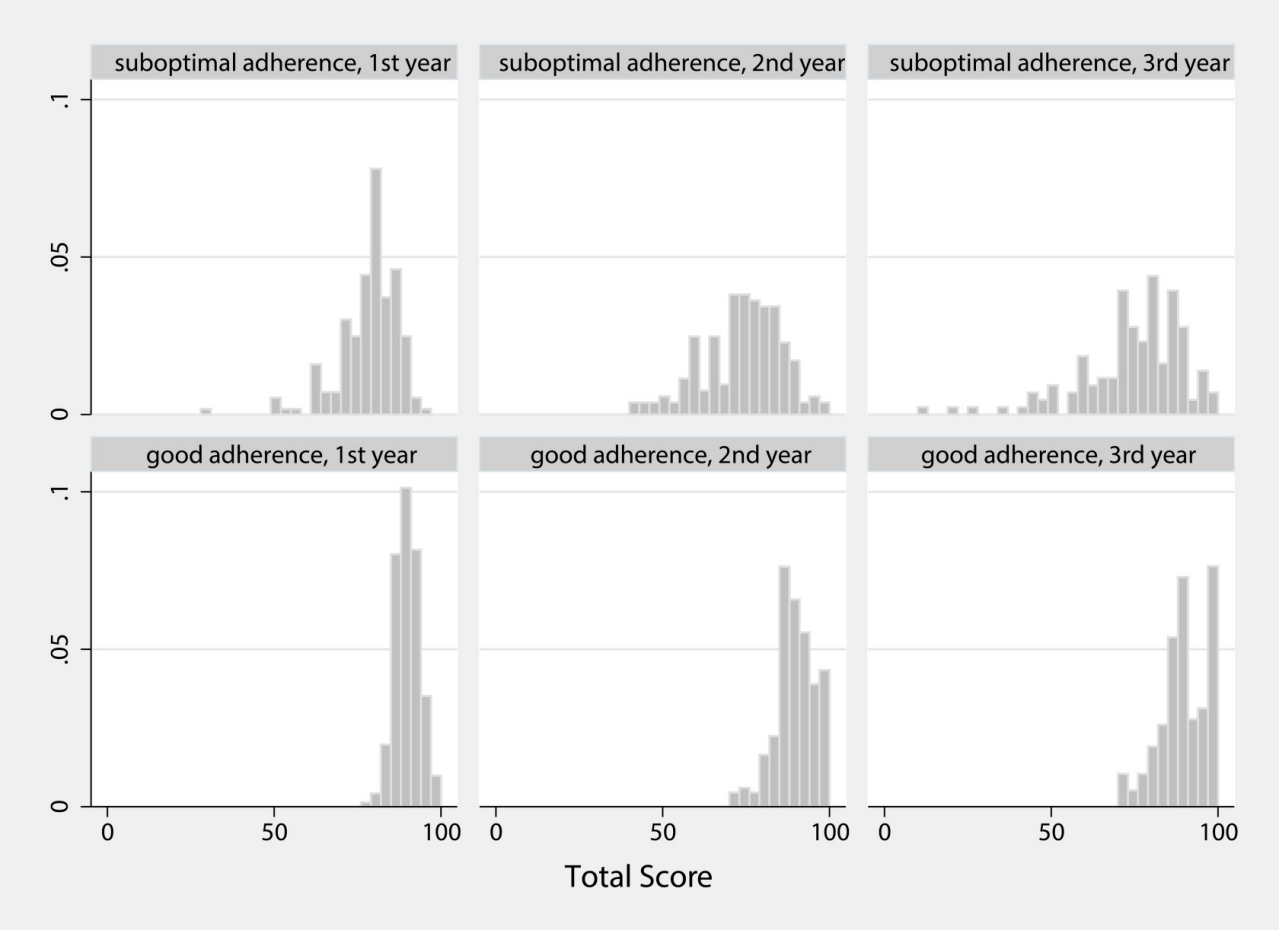
Good vs. suboptimal adherers over time. Histograms with the relative frequency (density) of combined adherence scores (x axis) for suboptimal vs. good adherers within the first three years after LTx. Patients with suboptimal adherence, i.e., below the median of 86% (upper panel), tended to a decline in their scores from year 1 to year 3. Patients with good adherence (lower panel) tended to keep their level.

ROC curves showed no satisfying results to distinguish between good and suboptimal adherers. The areas under the curve never exceeded 0.6, and no statistical significance was reached. Using the cut-off values of ROC curves as discriminators resulted in uneven groups and unsatisfying sensitivity and specificity. The respective findings for the mean 1–3 years adherence score were a threshold of 84.6% with a sensitivity of 62.8% and a specificity of 56.1% (Youden-Index of 0.189). Because of this limitation we decided to use the median instead as it divides the cohort into two even groups. Descriptive control of the median showed adequate discrimination. This resulted in a score of 86% within the first three years after LTx as a cut-off value to compare clinical outcome parameters. Patients with a mean adherence score below the median tended to lower scores over the three years, while patients with a mean score above the median mostly stayed above the median (Fig 2). In more detail, 85% of the patients remained good or suboptimal adherer, while only 9% changed from good to suboptimal and 6% from suboptimal to good adherence.

### Clinical outcome

**Fig 3:**
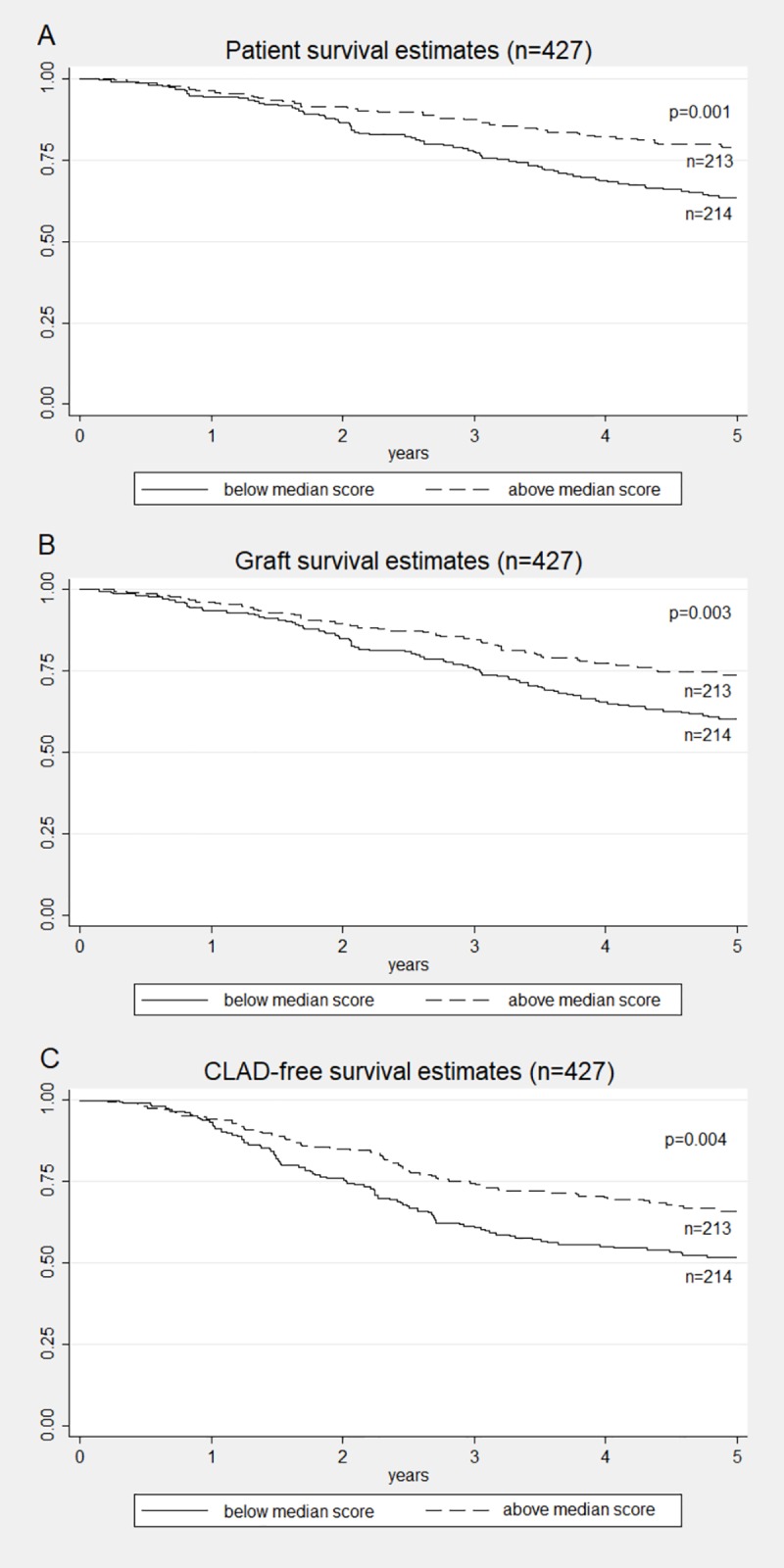
Survival estimates comparisons by adherence score cut-off. Kaplan-Meier estimates for 5 year (A) patient survival, (B) allograft survival, and (C) CLAD-free survival for patients with a 1–3 years good (dashed line) or suboptimal adherence (continuous line).

Kaplan-Meier estimates demonstrated better patient (Fig 3A), allograft (Fig 3B) and CLAD-free survival (Fig 3C) for patients with a good (i.e., above the median of 86%) 3 year mean adherence. Graft loss, CLAD and death occurred less frequently in good adherers (Table 3). We did the analysis both for the whole cohort and after exclusion of heart lung transplant patients. The latter did not change the results for the discriminating median (86.2%) and for the Kaplan-Meier estimates, although heart lung transplant patients were mostly in the suboptimal adherence group (12/13). That is most probably due to the low number of heart lung transplant patients.

**Table 3:** Clinical outcome.

	Good adherence n = 214 (50%)	Suboptimal adherence n = 213 (50%)	p-value
**Graft loss** n (%)	56 (26%)	85 (40%)	0.003
**Death**	45 (21%)	78 (36%)	<0.001
**CLAD (all forms)**	70 (33%)	96 (45%)	0.011
**RAS**	15 (7%)	22 (10%)	0.234
**Follow-up, years** Median (IQR)	6.1 (5.1–7.4)	5.7 (3.2–7.2)	0.009
**A1 or higher biopsy within first year** N (%)	69 (32%)	65 (30%)	0.653
**GFR at 1^st^ year** Mean (± SD)	74 (±21)	69 (±23)	0.013
**GFR at 3^rd^ year** Mean (± SD)	71 (±23)	64 (±24)	0.017
**GFR at 5^th^ year** Mean (± SD)	64 (±26)	58 (±27)	0.074
**Hospitalizations 1^st^ year** Mean (± SD)	1.6 (±1.9)	1.9 (±2.1)	0.025
**Days of Hospitalization 1^st^ year** Mean (± SD)	12 (±29)	17 (±33)	0.015
**QoL VAS 1^st^ year** Median (IQR)	7.7 (6.7–8.6)	7.5 (6.3–8.4)	0.118
**QoL VAS 2^nd^ year** Median (IQR)	8.0 (7.0–9.0)	7.6 (6.0–8.8)	0.016
**QoL VAS 3^rd^ year** Median (IQR)	8.1 (6.7–9.0)	7.0 (5.1–8.7)	< 0.001
**Visits outpatient 1^st^-3^rd^ year** Mean (± SD)	16.3 (±6.4)	14.7 (±7.7)	0.025
**Telephone contacts 1^st^-3^rd^ year** Mean (± SD) Mean (± SD)	5 (±4)	6 (±6)	0.265

All numeric variables are shown as median with interquartile range (IQR) or mean ± standard deviation (SD); all categorical variables are shown as n (%). GFR–glomerular filtration rate; QoL–quality of life. CLAD–chronic lung allograft dysfunction, BOS–bronchiolitis obliterans syndrome, RAS–restrictive allograft syndrome.

Survival curves showed best results for the 9% of patients that improved their adherence, and second best for those who always had good adherence. Those patients with suboptimal adherence and those who changed from good to suboptimal adherence had a worse outcome. However, the number of patients in the change-groups is too low to obtain valid statistical results.

There was no difference for the incidence of RAS or acute rejections (A1 or higher in biopsy) within the first year. Patients with suboptimal adherence had more and longer hospitalizations than good adherers, but visited the outpatient clinic less frequently. Renal function declined over time with a trend to a better course for patients with good adherence. Assessment of quality of life showed increasingly better values for the good adherence group from the first to the third year, while the low adherence group tended to lower EQ-5D scores within time (Table 3).

Patient survival tended to be associated with good adherence for all subscores (Table 4) with statistical significance for “trough levels” and “nutrition/exercise”. Graft survival tended to be better for good adherence in all subscores except “health perception”. Statistical significance was reached for “trough levels” and “nutrition/exercise”. In multivariate logistic regression, only nonadherence for the subscore “home spirometry” showed a significant effect on patient survival with an Odd’s ratio of 4.2 (p = 0.008) for a negative outcome. Since the regression coefficients for all subscores including “home spirometry” were approximately 1, the weights of the subscores were not changed for the composite score.

**Table 4:** Subscores 1–3 years after LTx.

	Median	Patient survival Below/above median	Allograft survival
**Subscore 01 *(health perception)***	91	69/54; p = 0.142	74/67; p = 0.550
**Subscore 02 *(home monitoring*, *frequency)***	98	70/53; p = 0.077	79/62; p = 0.093
**Subscore 03 *(contact)***	95	68/55; p = 0.187	76/65; p = 0.290
**Subscore 04 *(nutrition*, *exercise)***	83	77/46; p = 0.004*	86/55; p = 0.007*
**Subscore 05 *(trough levels)***	68	73/50; p = 0.013*	83/58; p = 0.010*

Patient survival tended to be better in patients who reached higher levels than the respective median for all individual subscores. Number of events for adherence below and above median are shown for patient and for allograft survivals. P-values show levels of significance (*).

### Disease specific differences

**Fig 4:**
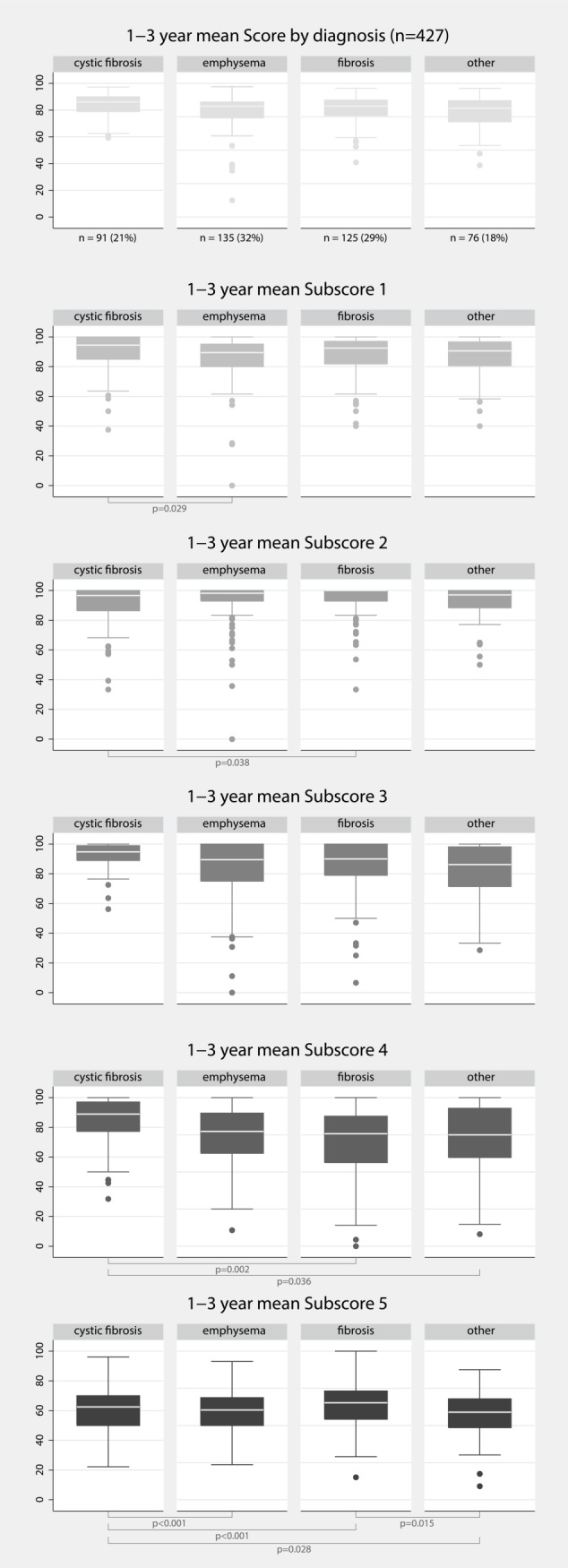
Adherence score and subscore comparisons by diagnosis group. Median combined adherence score and median subscores 1 (health perception), 2 (home monitoring), 3 (contact), 4 (nutrition, exercise), and 5 (trough levels) by diagnosis group. Medians are shown with IQR and outliers.

Demographics did not significantly differ between the two adherence groups (Table 2). Subscore analysis revealed a potential influence of primary disease on certain aspects of adherence (Fig 4). Health perception was impaired in emphysema compared to CF patients. Patients with pulmonary fibrosis used home spirometry more frequently than patients with CF. Regarding nutrition/exercise, CF patients showed better results than the others, but had more difficulties to perceive control of trough levels.

## Discussion

In this study, a good adherence based on objective health care worker judgement within the first 3 years after LTx was associated with better patient and graft survival and a lower incidence of CLAD.

Non-adherence is a major and long-known problem in medicine. The association between non-adherence to immunosuppressants and impaired outcome in solid organ transplantation seems obvious. As an accurate measure of adherence is not available, evidence for this association is scanty.

MEMS[18,19] are considered the gold standard to assess adherence to immunosuppressants in clinical studies[20]. Bosma and colleagues assessed adherence in LTx recipients using MEMS (median timing adherence 98.1%, range 31.2–100%), but did not report clinical outcome[18]. For heart transplant patients, non-adherence assessed by MEMS was associated with unfavorable outcomes[21]. However, there are important concerns on using MEMS to assess adherence. First, MEMS only cover adherence to the regular and timely intake of medication and no other aspect of adherence. Second, due to high costs and unpractical handling MEMS are not suitable for assessing adherence in a large number of patients in the clinical routine as in our retrospective study. Most importantly, MEMS by themselves are interventional tools and can therefore not be considered a real gold standard for the measurement of adherence.

Advanced electronic systems integrate interventions to improve adherence, such as alarms as a reminder to take the medication[22]. Ingestible sensors combined with or embedded in tablets may accurately determine taking adherence[23,24]. Recently, a necklace has been described that detects swallowing of tablets based on neck movements[25]. Whether or not those new devices will be introduced into clinical routine remains open.

Adherence can further be assessed by patients’ self-reports, e.g. the validated questionnaire BAASIS©[26]. In our transplant center, LTx and kidney transplant patients judged their adherence as poorer than health care professionals’ estimates, with our adherence score being an independent predictor of self-reported non-adherence in LTx patients[8,27]. MEMS results have been compared to patients’ and clinicians’ reports with weak to moderate correlation[28–30]. Patients tend to overestimate their adherence in self-reports with a self-reported adherence rate of 75–90%, while electronic measurements indicated adherence in less than 60% of patients[30]. Although the potential negative impact of non-adherence primarily affects themselves, they try to hide non-adherent behavior. Hypertensive patients were shown to take their medication more frequently 1–3 days before their clinic appointment[31]. Dobbels and colleagues reported an association between self-reported non-adherence (in 40% of participants) in the pre-transplantation period and late acute rejection for patients after lung, heart or liver transplantation. Pre-transplantation non-adherence predicted post-transplantation non-adherence, but the correlation between post-transplantation non-adherence and outcome was not analyzed[32,33]. In our work, the incidence of acute rejection was not associated with adherence. Thirty-two percent of patients had at least one A1 rejection in surveillance bronchoscopies. High grade acute rejection episode (≥ A2) were rare. It was demonstrated that low grade A1 rejection was not associated with CLAD[34]. Other groups used the discrepancy between immunosuppressant dosages and prescription refills or data from health insurances for the assessment of adherence[35,36]. Few publications have identified an association of non-adherence in the patient’s record to impaired graft survival[3,5]. In a recent registry analysis of 7,284 LTx recipients, 4-year non-adherence regarding immunosuppression was found in 10.6% of patients and was associated with shorter unadjusted survival[11]. No definitions for adherence were given and answers were limited to yes/no in this analysis.

Blood level variability of immunosuppressants is a widely used surrogate parameter of adherence. High variability was associated with unfavorable allograft outcome in solid organ transplant recipients, including patients after lung transplantation[10,37–39].

De Geest’s group suggested a combined assessment of patients’ self-reports, clinicians’ estimates, and blood levels[40]. Other groups[41–43] suggested that in addition to medication adherence, other dimensions, such as health perception, diet, exercise, and keeping appointments and contact with the transplant center might influence outcome. In this study, an effect on outcome was too small to be statistically significant for most categories, supporting the idea of a multidimensional adherence judgement. The subscores “trough levels” and “nutrition/exercise” seem to be the strongest in our score.

The association between immunosuppressant trough levels and allograft survival has been described before. Gallagher et al.[39] could demonstrate that higher mean tacrolimus trough levels 6–12 months after LTx were associated with reduced risk of CLAD but also that higher standard deviations of tacrolimus levels correlated with the incidence of CLAD and death. In line with this observation[10,37–39,44], we included trough levels by percentage of measurements in target range as a clinically practicable surrogate parameter. Trough levels may also be influenced by introduction or discontinuation of other medication with Azols being the most important ones. Azols are routinely used for all patients in our program and continued life-long. Even though a high trough level variability may not always be caused by non-adherence, it is important to recognize and identify potential mistakes. Low CNI levels may lead to under-immunosuppression and subsequently to rejections and CLAD. On the other hand, especially prolonged elevated CNI levels may be associated with infections, malignancies, kidney and vascular disease and thereby influence outcome.

In addition to regular physical exercise, BMI is the main factor for the subscore “nutrition/exercise”, and both relevant underweight and obesity translate into suboptimal scores. Although all our patients are required to have a normal BMI before transplantation, a pathological BMI after transplantation may not necessarily reflect non-adherence, especially in cases of malnutrition. An impact of both underweight and obesity on mortality has been reported for LTx recipients[45].

Regular physical exercise has a positive effect on quality of life, exercise capacity and post-transplant hypertension in LTx patients[46,47] and we encourage all our patients to regularly perform exercises by themselves or to take part in medical training programs, depending on their capabilities.

The purpose of daily home spirometry is the early detection of impaired allograft function. Patients are instructed to immediately seek contact with the transplant center in case of declining graft function. Regular home spirometry failed to reach a statistically significant association with patient and allograft survival in this study, which is in line with our former work that could only demonstrate an association with CLAD-free survival[12].

The subscores “health perception” and “contact” did not show a strong association to outcome. However, especially smoking has been associated with mortality, malignancies and cardiovascular disease after solid organ transplantation[48] and can objectively assessed by health care workers by monitoring blood or urine levels of cotinine.

Mean adherence scores declined with time after LTx, suggesting that patients tend to be more adherent in the first months after transplantation and to be more careless in the following years. This finding is in line with the literature on medication adherence in general[31] and in LTx patients[49] and was also demonstrated for self-reported adherence in patients from our center[27]. A possible explanation for this is that the patients are feeling better and try to live a normal life. Vanhoof and colleaques illustratively describe patients’ difficulties to implement the numerous rules for health behavior after transplantation into their daily routine[50]. We feel that it is crucial to recognize a decline in adherence in time in order to initiate measures to improve adherence, such as intensification of education[17] or implementation of applications for mobile devices[17,51,52].

Our study has several limitations. First, it is a single center study retrospectively analyzing adherence in the regular clinical setting. Interventions to improve adherence were performed and may have interfered. Secondly and importantly, not all events leading to an unfavorable score may have been a consequence of non-adherence: drug interactions or malabsorption can influence immunosuppressant trough levels, medical complications can make regular training difficult or lead to wasting and underweight. On the other hand, even in these cases our score is helpful to identify problems in order to offer solutions. Furthermore, our adherence score was not validated, which is due to the lack of a real gold standard or rather the considerable shortcomings of the MEMS as a “gold standard”. Finally, inter-observer variability was not tested, as the score is completed by the transplant coordinator, checked by the physician and discussed in team meetings. We tried to minimize subjective bias from health care workers by using objectively measurable and distinct items for the adherence score.

Our composite adherence score is a practical tool in every day clinical life, that can–with modifications–be transferred to other solid organ transplant recipients. For kidney transplant patients, for instance, home spirometry would have to be replaced by the regular self-measurement of blood pressure or body weight. The score can be used as an outcome measure in interventional studies on adherence, and factors leading to non-adherence (social, economic, health care team- and system-related factors, condition-, therapy- and patient-related factors[53]) can be further analyzed.

In summary, our multidimensional adherence score is a simple tool to assess adherence. We could demonstrate that good adherence assessed with this score was significantly associated with superior patient and allograft 5-year-survival after LTx, which could be explained by a lower incidence of CLAD. By using this score, suboptimal adherence can be recognized and measures to improve adherence can be taken in time.

## Supporting information

S1 File Anonymized data set. (CSV)

## Abbreviations

BMI: body mass index
BOS: bronchiolitis obliterans syndrome
CF: cystic fibrosis
CLAD: chronic lung allograft dysfunction
CNI: calcineurin inhibitor
FEV1: forced expiratory volume in 1 second
GFR: glomerular filtration rate
IQR: interquartile range
LTx: lung transplantation
MEMS: medication event monitoring system
QoL: quality of life
RAS: restrictive allograft syndrome
ROC: receiver operating characteristic
